# Copper-induced concomitant increases in photosynthesis, respiration, and C, N and S assimilation revealed by transcriptomic analyses in *Ulva compressa* (Chlorophyta)

**DOI:** 10.1186/s12870-019-2229-5

**Published:** 2020-01-15

**Authors:** Daniel Laporte, Felipe Rodríguez, Alberto González, Antonio Zúñiga, Eduardo Castro-Nallar, Claudio A. Sáez, Alejandra Moenne

**Affiliations:** 1 Laboratory of Marine Biotechnology, Faculty of Chemistry and Biology, University of Santiago of Chile, Alameda, 3363 Santiago, Chile; 2 HUB AMBIENTAL UPLA, Vicerrectoría de Investigación, Postgrado e Innovación, University of Playa Ancha, Avenida Carvallo 270, 2340000 Valparaíso, Chile; 3 Center of Bioinformatics and Integrative Biology, Faculty of Life Sciences, University Andrés Bello, República 330, Santiago, Chile; 4 Laboratory of Aquatic Environmental Research, Center of Advanced Studies, University of Playa Ancha, Traslaviña 450, Viña del Mar, Chile

**Keywords:** Copper, Photosynthesis, Respiration, Transcriptomic analyses, Marine alga, *Ulva compressa*

## Abstract

**Background:**

The marine alga *Ulva compressa* is the dominant species in copper-polluted coastal areas in northern Chile. It has been shown that the alga tolerates micromolar concentrations of copper and accumulates copper at the intracellular level. Transcriptomic analyses were performed using total RNA of the alga cultivated with 10 μ M copper for 0, 1, 3 and 5 days using RNA-seq in order to identify processes involved in copper tolerance.

**Results:**

The levels of transcripts encoding proteins belonging to Light Harvesting Complex II (LHCII), photosystem II (PSII), cytochrome b6f, PSI, LHCI, ATP synthase and proteins involved in repair of PSII and protection of PSI were increased in the alga cultivated with copper. In addition, the level of transcripts encoding proteins of mitochondrial electron transport chain, ATP synthase, and enzymes involved in C, N and S assimilation were also enhanced. The higher percentages of increase in the level of transcripts were mainly observed at days 3 and 5. In contrast, transcripts involved protein synthesis and degradation, signal transduction, and replication and DNA repair, were decreased. In addition, net photosynthesis and respiration increased in the alga cultivated with copper, mainly at days 1 to 3. Furthermore, the activities of enzymes involved in C, N and S assimilation, rubisco, glutamine synthase and cysteine synthase, respectively, were also increased, mainly at days 1 and 3.

**Conclusions:**

The marine alga *U. compressa* tolerates copper excess through a concomitant increase in expression of proteins involved in photosynthesis, respiration, and C, N and S assimilation, which represents an exceptional mechanism of copper tolerance.

## Background

In photosynthetic organisms, copper is an essential heavy metal that it is required for the activity of several proteins and enzymes such as plastocyanin, cytochrome c oxidase, Cu/Zn superoxide dismutase (SOD), polyphenol oxidase, laccase and ascorbate oxidase, among others [1, 2]. Copper is required only in trace amount as in excess it produces an oxidative stress condition that becomes harmful for cellular macromolecules [2]. In photosynthetic organisms, copper excess may lead to the replacement of magnesium in chlorophylls inhibiting the release of energy from chlorophylls to PSII under low light, or it can also directly inhibit the reaction center in PSII in under high light [3, 4]. Copper excess can also inactivate enzymes by replacing zinc or other heavy metals [5].

The marine green macroalgae *Ulva pertusa* and *U. armoricana* cultivated with increasing concentrations of copper, from 0 to 250 μg L^− 1^ (3.9 μM) for 3 days displayed a differential behavior regarding photosynthesis and N assimilation [6]. *U. pertusa* showed no inhibition of photosynthetic parameters until 100 μg L^− 1^ (1.5 μM) and an increase in the activity of nitrate reductase activity, an enzyme involved in N assimilation [6]. In contrast, *U. armoricana* showed inhibition of photosynthetic parameters at 50–100 μg L^− 1^ of copper and an inhibition of nitrate reductase activity [6]. The green macroalga *U. flexuosa* cultivated with 0.8, 4 and 8 μM of copper for 5 days showed inhibition of photosynthesis when cultivated with 4 and 8 μM copper [7]. *U. compressa* L. Grev. (Cholorophyta) showed an increase in photosynthesis when cultivated with 10 μM of copper for 0 to 24 h [8]. Thus, green macroalgae from the same genus displayed differential responses to copper stress and it seems that, among Ulvaceae, *U. compressa* is the most tolerant species regarding copper stress. The red macroalga *Gracilaria tenuistipitata* cultivated with 16 nM of copper for 1 to 6 days showed an inhibition of photosynthesis from day 1 [9]. The red macroalga *Porphyra haitiensis* cultivated with 0.1 to 50 μM of copper for 3 days showed an increase in photosynthesis at concentrations of 0.1 to 1 μM and an increase in respiration with 0.1 to 50 μM suggesting that respiration is less sensitive to copper stress [10]. The brown macroalga *Ectocarpus siliculosus* showed an increase in photosynthesis when cultivated with 1.8 μM of copper for 8 h but a decrease in photosynthesis with 3.7 μM of copper [11]. Thus, marine macroalgae showed differential behaviors regarding photosynthesis and respiration in response to copper stress.

The marine alga *U. compressa* is highly tolerant to copper excess since the alga cultivated with 50 μM copper for 7 days displayed cellular viability [12]. The alga exposed to a sub-lethal concentration of copper (10 μM) showed the accumulation of superoxide anions beginning at day 3 and increasing until day 7 and the production of superoxide anions occurred mainly in chloroplasts and mitochondria [12]. In addition, the alga cultivated with copper showed the activation of antioxidant enzymes such as superoxide dismutase, ascorbate peroxidase and glutathione reductase, and the synthesis of antioxidant compounds such as ascorbate (ASC) and glutathione (GSH) [12–14]. In addition, *U. compressa* accumulate copper in its tissue, reaching 620 μg g^− 1^ when cultivated with 10 μM copper for 12 days [15]. Copper accumulation correlates with the synthesis of GSH, and phytochelatins (PCs), which are peptides produced through condensation of GSH units, and with the increase in expression of metallothioneins (MTs), which are small size cysteine-rich proteins that bind monovalent and divalent metal ions [16]. Initial transcriptomic analyses using RNAseq performed in *U. compressa* cultivated with 10 μM copper for 0 and 24 h allowed the identification of 7 potential MTs as well as transcripts that encode antioxidant enzymes, and enzymes involved in ASC and GSH synthesis [16]. The levels of transcripts encoding MTs, antioxidant enzymes, and enzymes involved in ASC and GSH synthesis, were increased in the alga exposed to copper excess [16]. Finally, transcripts encoding three MTs, MT1, MT2 and MT3, were cloned from *U. compressa* and their overexpression in bacteria allowed the accumulation of copper and zinc in vivo [17]. Thus, *U. compressa* may accumulate copper in its tissue through the binding of copper ions to GSH, PCs and MTs and this alga could represent a useful tool for phycoremediation of seawater contaminated with heavy metals.

Photosynthetic organisms displaying an increase in photosynthesis may produce an enhanced level of NADPH that may increase C, N and S assimilation, since the latter are reductive processes that require NADPH [18–20]. The marine macroalga *U. compressa* cultivated with 10 μM copper for 0 to 24 h showed an increase in photosynthesis and in the level of transcripts encoding enzymes of the Calvin-Benson cycle, suggesting that C assimilation may be increased [8]. In this work, we investigate whether the initial increase in photosynthesis is maintained along days, and whether there is a concomitant increase in respiration, and in C, N and S assimilation. To this end, the alga *U. compressa* was cultivated with 10 μM copper for 0, 1, 3, and 5 days and the levels of transcripts encoding proteins involved in photosynthesis and respiration, and those encoding enzymes involved C, N and S assimilation, were determined. In addition, the level of produced and consumed oxygen, reflecting photosynthesis and respiration, respectively, were determined. The activities of key enzymes involved in C, N and S assimilation such as rubisco, glutamine synthase and cysteine synthase, respectively, were also analyzed. Results indicate that the alga tolerates copper excess through a concomitant increase in expression of proteins involved in photosynthesis, respiration, and C, N and S assimilation, which represents an exceptional mechanism of copper tolerance.

## Results

### Assembly, annotation and classification of transcripts

The libraries obtained from samples of the alga cultivated in control condition (day 0) and treated with copper for 1, 3, and 5 days contained 785,500,000 M of reads, in total, and 98,187,500 reads, on average. The reads were subjected to quality control, they were trimmed and bacterial sequences were eliminated, resulting in 96,577,500 M of reads, on average, which correspond to 98.36% of the initial reads (Additional file 1: Table S1). Transcripts were assembled using Trinity software and resulted in 140,840 transcripts (contigs) of 300 to 5140 nucleotides in length, with an average length of 1575 nucleotides. The completeness of assembled transcriptomes was 93.4% (Additional file 1: Table S1). Transcripts were translated into amino acids, and annotated proteins having an e value of 1e^− 3^, or lower, were 65,494 (Additional file 5: Table S2). The latter proteins are involved in different biological processes (Additional file 2: Figure S1). Of the 65,494 proteins, 23,692 (36%) showed higher amino acid similarity with plant and green algae (Plantae) proteins; 15,073 (23%) displayed a higher similarity with animal proteins; and 26,729 (41%) proteins showed similarity to other organisms (Fungi, Protista and Prokaryotes) (Additional file 3: Figure S2). Proteins having similarity to animal proteins showed homology with human, mouse, rat, and other animal proteins (Additional file 3: Figure S2).

### Transcripts differentially expressed in response to copper excess

Transcripts coding for 65,494 proteins previously mentioned that were differentially expressed were obtained considering times: 0 vs. 1, 0 vs. 3 and 0 vs. 5 (Additional file 4: Figure S3). At time point 0 vs. 1, the number of differentially expressed transcripts was 28,510, those up-regulated were 13,855 and those down-regulated were 14,655, which represent 48.6 and 51.4%, respectively. At time point 0 vs. 3, the number of differentially expressed transcripts was 8174, those up-regulated were 6047 and those down-regulated were 2127, which represent 74 and 26%, respectively. At time point 0 vs. 5, the number of differentially expressed transcripts was 30.589, those up-regulated were 17,218 and those down-regulated were 13,371, which represent 56.3 and 43.7%, respectively. Thus, the higher percentages of up-regulated transcripts were observed at days 3 and 5 in the alga exposed to copper excess.

### Transcripts with increased levels of encoded proteins involved in photosynthesis and respiration

Regarding photosynthesis, the levels of transcripts encoding subunits of PSII corresponding to PsbA, PsbB, PsbC, PsbE, PsbH, PsbN, PsbO, PsbP, PsbR, PsbS, PsbW, PsbY and PsbZ were up-regulated in response to copper stress (Table 1). In addition, the levels of transcripts encoding subunits of Light Harvesting Complex II (LHCII), chlorophyll a/b-binding proteins LhcB1, LhcB4, LhcB5 and Cab1 as well as fucoxanthin-chlorophyll a/c-binding proteins, FcpA and FcpB, were increased. In addition, the subunits of the enzyme magnesium chelatase involved in chlorophyll synthesis, ChlD and ChlI, were up-regulated (Table 1). The levels of transcripts encoding subunits of cytochrome b6f corresponding to cytochrome b6 (petB), iron-sulfur Rieske subunit (PetC), an essential protein for assembly of cytb6f (PetG), and a carrier of electrons from cytb6f to PSI (PetJ), were also increased (Table 1). The levels of transcripts encoding subunits of PSI corresponding to PsaA, PsaB, PsaD, PsaF, PsaG and PsaL were also up-regulated (Table 1). Finally, the levels of transcript encoding a subunit of LHCI, LhcA, and the subunits of ATP synthase α, β, γ, δ, ε and γ subunits were also increased (Table 1).

**Table 1:** Up-regulated genes related to photosynthesis and mitochondrial electron transport chain

Process	ID Transcript	Proteins	Log2 Fold Change
PSII	Unigene17497_All	PsbA	3.4
	CL2571.Contig1_All	PsbB	2.0
	CL11753.Contig1_All	PsbB	2.4
	CL11753.Contig2_All	PsbB	4.5
	Unigene33413_All	PsbC	3.3
	CL4160.Contig3_All	PsbE	2.6
	Unigene34274_All	PsbH	3.3
	Unigene29880_All	PsbN	3.3
	Unigene13168_All	PsbO	1.6
	Unigene29944_All	PsbP	2.1
	CL7636.Contig1_All	PsbP	1.2
	CL699.Contig2_All	PsbP	2.3
	CL4419.Contig1_All	PsbP	1.8
	CL4419.Contig5_All	PsbP	1.2
	CL3599.Contig1_All	PsbR	2.0
	CL10892.Contig2_All	PsbS	3.6
	Unigene33763_All	PsbW	5.3
	Unigene37851_All	PsbY	1.6
	Unigene1513_All	PsbZ	2.5
LHCII	Unigene596_All	Chlorophyll a-b binding protein 1D (lhcB1)	2.6
	CL4261.Contig2_All	Chlorophyll a-b binding protein CP26 (lhcB5)	1.4
	CL11211.Contig2_All	Chlorophyll a-b binding protein CP26 (lhcB5)	1.9
	CL1500.Contig3_All	Chlorophyll a-b binding protein L1818 (lhcb4)	4.3
	CL4399.Contig3_All	Chlorophyll a-b binding protein L1818 (lhcb4)	6.8
	CL9440.Contig1_All	Chlorophyll a-b binding protein L1818 (lhcb4)	3.0
	CL9440.Contig2_All	Chlorophyll a-b binding protein L1818 (lhcb4)	4.7
	Unigene29850_All	Chlorophyll a-b binding protein L1818 (lhcb4)	1.5
	CL1500.Contig2_All	Chlorophyll a-b binding protein L1818 (lhcb4)	2.5
	CL4399.Contig1_All	Chlorophyll a-b binding protein L1818 (lhcb4)	1.4
	CL12301.Contig27_All	Chlorophyll a-b binding protein Type I (CabII-1)	2.9
	CL12301.Contig28_All	Chlorophyll a-b binding protein Type I (CabII-1)	2.5
	Unigene22628_All	Fucoxanthin-chlorophyll a-c binding protein (FcpA)	1.0
	CL941.Contig8_All	Fucoxanthin-chlorophyll a-c binding protein (FcpE)	2.4
	CL2528.Contig4_All	Magnesium-chelatase subunit ChlD	1.8
Cyt b6-f	Unigene41646_All	Iron-sulfur subunit (petB)	1.8
	CL2893.Contig2_All	Iron-sulfur subunit (petC)	1.5
	CL2893.Contig4_All	Iron-sulfur subunit (petC)	3.7
	CL2893.Contig6_All	Iron-sulfur subunit (petC)	5.7
	CL2893.Contig9_All	Iron-sulfur subunit (petC)	1.1
	CL2893.Contig10_All	Iron-sulfur subunit (petC)	4.9
	Unigene174_All	Iron-sulfur subunit (petC)	2.5
	Unigene970_All	Iron-sulfur subunit (petG)	2.5
	Unigene33787_All	Iron-sulfur subunit (petJ)	2.1
PSI	Unigene61010_All	PsaA	7.7
	Unigene955_All	PsaD	1.6
	Unigene21099_All	PsaF	1.3
	Unigene38056_All	PsaG	3.3
	Unigene25315_All	PsaL	1.3
LHC I ATP synthase	CL11079.Contig2_All	Chlorophyll a-b binding protein 5 (LhcA1 like)	3.4
	Unigene33050_All	ATP synthase subunit alpha	2.8
	CL10342.Contig2_All	ATP synthase subunit alpha	2.6
	Unigene28940_All	ATP synthase subunit alpha	1.4
	Unigene21967_All	ATP synthase subunit alpha	2.7
	CL5620.Contig2_All	ATP synthase subunit alpha	2.8
	CL10342.Contig6_All	ATP synthase subunit alpha	1.4
	CL2062.Contig2_All	ATP synthase subunit beta	8.1
	Unigene284_All	ATP synthase subunit beta	1.3
	Unigene283_All	ATP synthase subunit beta	1.9
	Unigene286_All	ATP synthase subunit beta	4.1
	CL2062.Contig1_All	ATP synthase subunit beta	1.2
	Unigene33306_All	ATP synthase subunit beta	2.1
	CL4160.Contig1_All	ATP synthase subunit beta	3.9
	Unigene19914_All	ATP synthase subunit beta	2.4
	CL5278.Contig5_All	ATP synthase subunit gamma	1.4
	Unigene16523_All	ATP synthase subunit gamma	2.2
	CL5278.Contig5_All	ATP synthase subunit gamma	1.4
	CL5278.Contig10_All	ATP synthase subunit gamma	1.4
	Unigene16523_All	ATP synthase subunit gamma	2.2
	Unigene32932_All	ATP synthase subunit epsilon	1.0
	Unigene43520_All	ATP synthase subunit delta	2.4
Assembly and Repair of PS II	Unigene29539_All	Met1	5.6
	CL1106.Contig1_All	Deg/HtrA Protease Do-like 1	4.2
	CL1106.Contig2_All	Deg/HtrA Protease Do-like 1	2.6
	CL1106.Contig5_All	Deg/HtrA Protease Do-like 1	4.5
	CL1106.Contig7_All	Deg/HtrA Protease Do-like 1	2.1
	CL1106.Contig8_All	Deg/HtrA Protease Do-like 1	2.5
	CL1106.Contig9_All	Deg/HtrA Protease Do-like 1	2.4
	CL1106.Contig13_All	Deg/HtrA Protease Do-like 1	2.6
	CL3921.Contig1_All	Deg/HtrA Protease Do-like 1	3.6
	CL3921.Contig3_All	Deg/HtrA Protease Do-like 1	2.0
	CL6287.Contig3_All	Deg/HtrA Protease Do-like 2	1.4
	CL6287.Contig4_All	Deg/HtrA Protease Do-like 2	1.0
	Unigene12439_All	Deg/HtrA Protease Do-like 9	1.0
	Unigene12443_All	Deg/HtrA Protease Do-like 9	1.1
	Unigene7791_All	ATP-dependent zinc metalloprotease FtsH1	1.5
	Unigene9549_All	ATP-dependent zinc metalloprotease FtsH1	1.1
	Unigene71521_All	ATP-dependent zinc metalloprotease FtsH1	3.1
	Unigene36205_All	ATP-dependent zinc metalloprotease FtsH1	9.1
	CL8065.Contig1_All	ATP-dependent zinc metalloprotease FtsH2	2.5
	Unigene16464_All	ATP-dependent zinc metalloprotease FtsH11	2.1
	CL6915.Contig1_All	ABC1K1	3.2
	CL6915.Contig7_All	ABC1K1	3.8
	CL12141.Contig2_All	2-carboxy-1,4-naphthoquinone phytyltransferase	2.3
	CL12141.Contig5_All	2-carboxy-1,4-naphthoquinone phytyltransferase	2.1
Assembly and Protection PSI	Unigene778_All	PGR5 1A	6.4
	CL653.Contig10_All	Serine/threonine-protein kinase STN8	3.1
	CL653.Contig13_All	Serine/threonine-protein kinase STN8	3.2
	CL653.Contig38_All	Serine/threonine-protein kinase STN8	2.9
	Unigene13129_All	YCF12	3.1
	CL5916.Contig1_All	ATAB2	3.0
	CL5916.Contig2_All	ATAB2	3.6
Mitochondrial	CL2273.Contig1_All	NADH dehydrogenase subunit 1	3.2
Electron	Unigene75664_All	NADH dehydrogenase subunit 2	3.1
Transport	CL8189.Contig1_All	cytochrome bc1 complex subunit V	2.4
Chain	CL5196.Contig1_All	Cytochrome bc1 complex subunit IV	2.8
	Unigene965_All	Cytochrome bc1 complex subunit IV	3.1
	Unigene30266_All	ATP synthase subunit gamma	3.1

On the other hand, the levels of transcripts encoding the chaperone MET1, involved in the insertion of PsbA (D1) in PSII; the ATP-independent serine proteases Deg 1, 2 and 9, and the ATP-dependent metalloproteases FtsH 1, 2 and 11, involved in degradation of damaged PsbA, were increased (Table 2). The levels of transcripts encoding bc1 complex kinase 1 (ABC1K1), involved in the synthesis of quinones and tocopherol (vitamin E) and the xanthophyll lutein which protect PSII to oxidative damage; the enzyme 2-carboxy-1,4-naphtoquinone phytyl transferase (ABC4), involved in the synthesis of phylloquinone (vitamine K) required in PSI, were up-regulated (Table 1). Moreover, transcripts encoding PGR5-1A, a protein involved in control of electron flow around PSI that protects PSI against photo-oxidation; YCF12, a protein involved in assembly and stabilization of PSI; the serine-threonine kinase STN8, involved in phosphorylation of LHCII allowing migration of subunits of PSII to PSI; and ATAB2, a light-regulated protein involved in the increased synthesis of photosystem proteins, were also increased (Table 1). Thus, the expression of a large number of proteins involved in photosynthesis and repair and protection of photosystems was increased in *U. compressa* exposed to copper excess (Additional file 6: Figure S4A).

**Table 2:** Up-regulated genes related to Carbon, Nitrogen and Sulfur assimilation

	ID Transcript	Proteins	Log2 Fold Change
Calvin-Benson Cycle	CL5634.Contig3_All	Ribulose bisphosphate carboxylase small chain 1	1.2
	CL5634.Contig2_All	Ribulose bisphosphate carboxylase small chain 1	8.9
	CL12005.Contig2_All	Ribulose bisphosphate carboxylase small chain 1	4.7
	CL12005.Contig1_All	Ribulose bisphosphate carboxylase small chain 1	1.4
	Unigene16805_All	Ribulose bisphosphate carboxylase small chain 1	2.8
	CL5634.Contig4_All	Ribulose bisphosphate carboxylase small chain 1	1.3
	CL12005.Contig3_All	Ribulose bisphosphate carboxylase small chain 2	1.8
	Unigene12933_All	Ribulose bisphosphate carboxylase large chain	1.6
	CL8954.Contig2_All	Phosphoglycerate kinase	2.1
	Unigene41371_All	Phosphoglycerate kinase	3.4
	CL1769.Contig1_All	Phosphoglycerate kinase	1.0
	CL1769.Contig2_All	Phosphoglycerate kinase	1.5
	Unigene32099_All	Phosphoglycerate kinase	4.7
	Unigene41512_All	Phosphoglycerate kinase	5.1
	CL1973.Contig6_All	Glyceraldehyde-3-phosphate dehydrogenase	6.0
	CL1973.Contig3_All	Glyceraldehyde-3-phosphate dehydrogenase	1.3
	CL8458.Contig1_All	Glyceraldehyde-3-phosphate dehydrogenase	2.5
	CL8458.Contig2_All	Glyceraldehyde-3-phosphate dehydrogenase	3.0
	CL1973.Contig7_All	Glyceraldehyde-3-phosphate dehydrogenase	1.0
	CL12146.Contig6_All	Glyceraldehyde-3-phosphate dehydrogenase	1.6
	Unigene26_All	Glyceraldehyde-3-phosphate dehydrogenase	1.5
	Unigene32957_All	Fructose-bisphosphate aldolase 1	7.0
	Unigene32956_All	Fructose-bisphosphate aldolase 1	2.5
	CL2940.Contig6_All	Fructose-bisphosphate aldolase 1	1.3
	Unigene13983_All	Fructose-bisphosphate aldolase 5	3.8
	CL2275.Contig2_All	Fructose-bisphosphate aldolase 8	1.0
	Unigene29154_All	Fructose-1,6-bisphosphatase	1.3
	Unigene38319_All	Transketolase	4.3
	Unigene29707_All	Transketolase	1.8
	CL11843.Contig2_All	Phosphoribulokinase	1.9
	Unigene42641_All	Phosphoribulokinase	2.9
Nitrogen Assimilation	Unigene26010_All	Nitrate reductase	2.4
	CL10485.Contig1_All	Nitrate reductase	3.0
	Unigene32044_All	Glutamine synthetase	5.3
	Unigene79885_All	Glutamine synthetase	1.2
	Unigene10873_All	Glutamine synthetase	2.3
	CL1194.Contig12_All	Argininosuccinate lyase	3.7
	CL1194.Contig11_All	Argininosuccinate lyase	9.2
	CL1194.Contig13_All	Argininosuccinate lyase	1.3
	Unigene25622_All	Fumarate hydratase	4.1
	Unigene14757_All	Fumarate hydratase	1.1
Sulfur assimilation	CL1413.Contig2_All	ATP sulfurylase	1.8
	CL1413.Contig5_All	ATP sulfurylase	1.2
	CL1413.Contig8_All	ATP sulfurylase	1.9
	CL1413.Contig9_All	ATP sulfurylase	2.7
	Unigene66570_All	ATP sulfurylase	3.1
	Unigene1609_All	APS reductase	1.8
	CL7094.Contig2_All	APS reductase	3.3
	Unigene803_All	APS reductase	1.9
	Unigene15137_All	APS reductase	5.4
	Unigene17508_All	Sulfite reductase	1.7
	CL9121.Contig1_All	Sulfite reductase	2.4
	CL9650.Contig2_All	Sulfite reductase	2.8
	Unigene823_All	Cysteine synthase	2.1
	CL10220.Contig15_All	Cysteine synthase	3.1
	CL10220.Contig39_All	Cysteine synthase	2.2
	CL10220.Contig51_All	Cysteine synthase	1.9
	CL10220.Contig56_All	Cysteine synthase	1.0
	Unigene33426_All	Cysteine synthase	3.9
	Unigene1587_All	Cysteine synthase	4.6
	CL1730.Contig3_All	Glutamate-cysteine ligase	2.1
	CL2777.Contig3_All	Glutamate-cysteine ligase	1.8
	Unigene6218_All	Glutamate-cysteine ligase	4.2
	Unigene6958_All	Glutamate-cysteine ligase	2.6
	Unigene5542_All	Glutamate-cysteine ligase	3.6
	Unigene25762_All	Glutamate-cysteine ligase	1.2
	Unigene80578_All	Glutamate-cysteine ligase	5.4
	Unigene28997_All	Glutathione synthetase	3.6
	Unigene28998_All	Glutathione synthetase	1.3
	Unigene28999_All	Glutathione synthetase	1.8
	Unigene42348_All	Glutathione synthetase	1.3

Regarding respiration, the levels of transcripts encoding subunit 1 and 2 of NADH dehydrogenase (complex I), subunit IV and V of cytochrome bc1 complex (complex III), and subunit γ of mitochondrial ATPase were increased (Table 1). Thus, the expression of a small number of proteins involved in respiration was increased in *U. compressa* compare to those involved in photosynthesis (Additional file 6: Figure S4B).

### Transcripts with increased levels of encoded enzymes involved in C, N and S assimilation

The levels of transcripts encoding enzymes of the Calvin-Benson cycle involved in C assimilation, RbcS and RbcL corresponding to the small and large subunits of rubisco; phosphoglycerate kinase (PGK); glyceraldehyde 3-P dehydrogenase (G3PDH); fructose biphosphate aldolase (FBPA); fructose 1,6 biphosphatase (FBP), transketolase (TK); ribose 5-P isomerase (R5PI) and phosphoribulose kinase (PRK) were increased (Table 2). Thus, the expression of nine enzymes of the eleven enzymes of the Calvin-Benson cycle was increased in *U. compressa* exposed to copper excess.

The levels of transcripts encoding enzymes involved in N assimilation, nitrate reductase and glutamine synthase, were increased (Table 2). In addition, the levels of transcripts encoding enzymes of the urea cycle allowing detoxification of ammonium excess such as arginino-succinate lyase and fumarate hydratase were up-regulated (Table 2). Thus, the expression of two of the three enzymes involved in N assimilation was increased in *U. compressa* exposed to copper excess.

The levels of transcripts encoding enzymes involved in S assimilation, adenylyl-sulfate transferase (ATP sulfurylase); adenosine 5′-phosphosulfate reductase (APS reductase); sulfite reductase, and cysteine synthase (O-acetylserine thiol lyase) were increased (Table 2). In addition, enzymes involved in the synthesis of amino acids methionine, serine and alanine were also increased (data not shown). Furthermore, the levels of transcripts encoding enzymes involved in glutathione synthesis, glutamate cysteine ligase (γ-glutamyl cysteinyl synthase) and glutathione synthase, were also up-regulated (Table 2). Thus, the expression of the four enzymes involved in S assimilation was increased in *U. compressa* exposed to copper excess.

### Kinetics of normalized reads of transcripts involved in photosynthesis and respiration

**Fig. 1:**
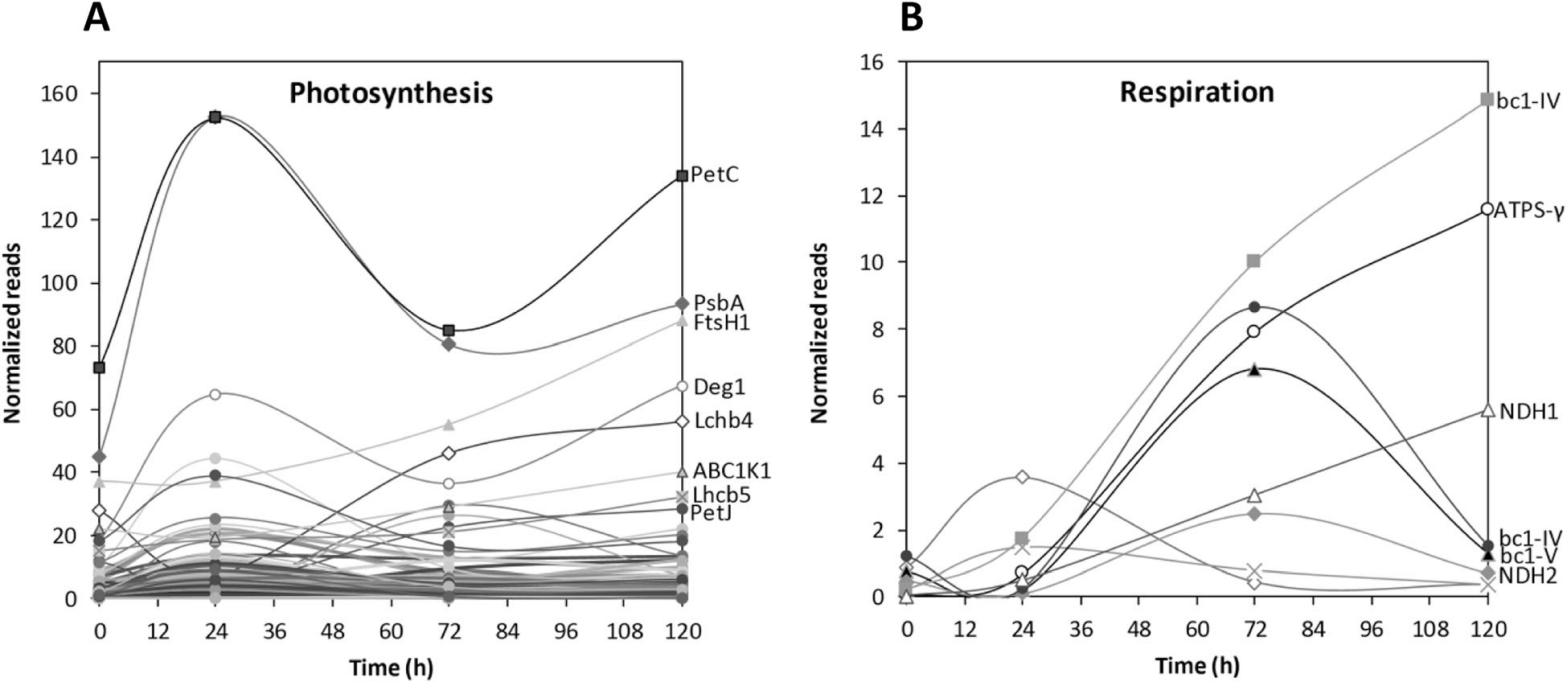
Level of the highest increased transcripts encoding proteins involved in photosynthesis (**a**) and respiration (**b**) in *U. compressa* cultivated with 10 μM copper for 0, 1, 3 and 5 days. Transcripts encoding proteins involved in photosynthesis corresponding to subunit PetC of cytb6f complex (PetC), subunit PsbA of PSII (PsbA), the protease FstH1 (FstH1), the protease Deg1 (Deg1), subunit 4 of LHCII (Lhcb4), the kinase ABC1K1, subunit 5 of LHCII (Lhcb1) and subunit PetJ of cytb6f complex (PetJ) are indicated with an arrow (**a**). Transcripts encoding proteins of the mitochondrial electron transport chain (respiration) corresponding to subunit IV of bc1 complex (complex III), subunit γ of ATP synthase (ATP-γ), subunit 1 of NADH dehydrogenase (complex I), subunit V of bc1 complex (complex III) and subunit 2 of NADH dehydrogenase (complex I) are indicated with an arrow (**b**). The level of transcripts is expressed as the number of normalized reads and time in days

The levels of normalized reads of transcripts encoding proteins involved in photosynthesis that showed the highest increases were: subunit PetC of cytb6f that increased at day 1, decreased at day 3, and increased again at day 5; subunit PsbA of PSII that increased at day 1, decreased at day 3, and slightly increased at day 5; protease FtsH1 that increased from day 3 to day 5; proteases Deg1 that increased at day 1, decreased at day 3, and increased again at day 5; subunits LhB4 and LhB5 of LHCII that increased at day 3 and remained increased until day 5; subunit PetJ of cytb6f and the kinase ABC1K1 that increased at day 1, decreased at day 3 and remained decreased at day 5 (Fig. 1a).

The levels of normalized transcripts encoding subunits of mitochondrial electron chain that showed the highest increases were: cytochrome c1 (subunit IV of complex III); subunit γ of ATP synthase and subunit 1 of NADH dehydrogenase (complex I) that increased at day 3 and remained increased until day 5; cytochrome c1 (subunit IV of complex III) and subunit γ of ATP synthase that increased at day 3 and decreased at day 5; subunit V of complex III and subunit 2 of NADH dehydrogenase that increased at day 1, decreased at day 3, and remained decreased at day 5 (Fig. 1b).

### Kinetics of normalized reads of transcripts involved in C, N and S assimilation

**Fig. 2:**
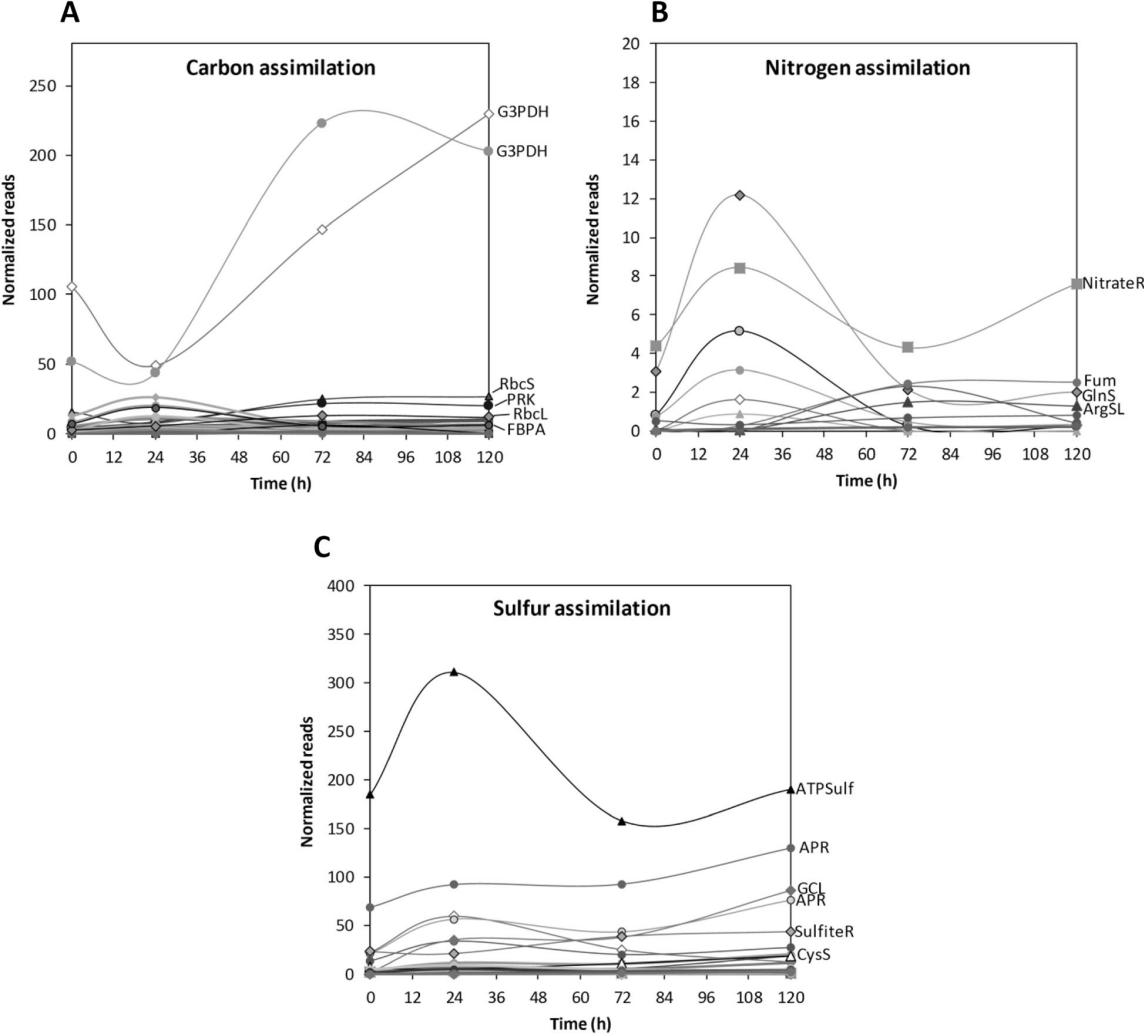
Level of the highest increased transcripts encoding enzymes involved in C, N and S assimilation in *U. compressa* cultivated with 10 μM copper for 0, 1, 3 and 5 days. Transcripts encoding enzymes involved in C assimuilation corresponding to glyceraldehyde 3-P dehydrogenase (G3PDH), small subunit of rubisco (RbcL), phosphorribulo kinase (PRK), large subunit of rubisco (RbcL) and fructose biphosphate aldolase (FBPA) are indicated with an arrow (**a**). Transcripts encoding enzymes involved in N assimilation corresponding to nitrate reductase (NitrateR), fumarase (Fum), glutamine synthase (GlnS) and arginine-succinate lyase (ArgSL) are indicate with an arrow (**b**). Transcripts encoding enzymes involved in S assimilation corresponding to ATP sulfurylase (ATPS), APS reductase (APSR) glutamine cysteine ligase (GCL), sulfite reductase (SulfiteR) and cysteine synthase are indicated with an arrow (**c**). The level of transcripts is expressed as the number of normalized reads and time in days

The levels of normalized reads of transcripts encoding enzymes involved in C assimilation that showed the highest increases were: G3PDH that increased mainly at day 3, and remained increased until day 5; rubisco small subunit RbcS, rubisco large subunit RbcL and enzymes PRK and FBPA that increased at day 3 and remained increased until day 5 (Fig. 2a).

The levels of normalized reads of transcripts encoding enzymes involved in N assimilation that showed the highest increases were: nitrate reductase that increased day 1, decrease at day 3, and increased again at day 5; glutamine synthase that increased at day 1, decreased at day 3, and remained decreased at day 5; fumarate hydratase and argino-succinate lyase that increased at day 3 and remained increased until day 5 (Fig. 2b).

The levels of normalized reads of transcripts encoding enzymes involved in S assimilation showing the highest increases were: ATP sulfurylase that increased at day 1, decreased at day 3, and slightly increased at day 5; APS reductase that increased at day 3 and continued to increase until day 5; glutamine cysteine ligase and APR reductase that increased at day 3 and remained increased until day 5; sulfite reductase that increased at day 1 and remained increased at days 3 and 5; and cysteine synthase that increased at day 3 and remained increased at day 5 (Fig. 2c). It is important to mention that the number of normalized reads of transcripts encoding enzymes of C and S assimilation showed higher levels (20 to 300 reads) compare to those of enzymes involved in N assimilation (1–12 reads).

### Transcripts with decreased levels encode proteins involved in protein synthesis and degradation, signal transduction, and replication and DNA repair

The most down-regulated transcripts at time points 0 vs. 1, 0 vs. 3 and 0 vs. 5 were involved in protein synthesis and degradation, signal transduction and, replication and DNA repair. Transcripts encoding proteins involved in protein synthesis and degradation were: ribosomal proteins L2, L15, L16, S12 and S17, ribosomal protein S6 kinase, eukaryotic translation factor 5B, tRNA dehydrouridine synthase, elongation factor 2a kinase, cysteine tRNA ligase, glu-tRNA amidotransferase, peptidyl prolyl trans-isomerase CYP61, RING finger protein 32, protease ESD4, neurotrypsin, E3 ubiquitin protein ligase, signal peptide peptidase, prefoldin subunit 6, Hsp40 (DNAJ) and hsp70, among others (Additional file 5: Table S2). Transcripts encoding proteins involved in signal transduction were: calreticulin, the serine/threonine protein kinases PKWA, PRP4, SAPK8 and SAPK38, the MAPKKK11, myb-related protein 1, tyrosine protein kinase SRK3, the protein phosphatases 1 regulator subunit 7 and the bifunctional phophatase IMPL2, among others (Additional file 5: Table S2). Transcripts encoding proteins involved in replication and DNA repair were: histone deacetylase HD11, DNA polymerase *ε* subunit subunit B, DNA topoisomerase 6 subunit A, DNA helicase II, RNA helicase DExH10, RNA polymerase I subunit RPA12, DNA repair protein RAD45, and DNA mismatch repair protein MSH13, among others (Additional file 5: Table S2).

### Copper-induced increases in net photosynthesis and respiration

**Fig. 3:**
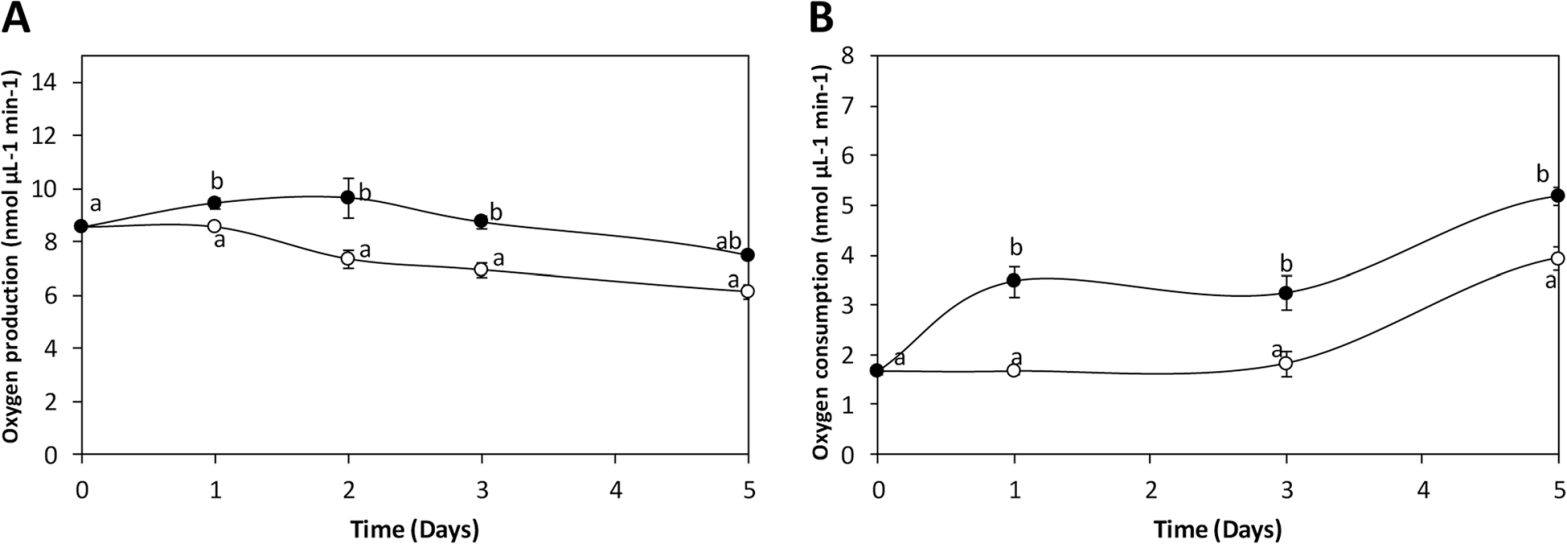
Level of oxygen production under light (**a**) and oxygen consumption in the dark (**b**) in the marine alga *U. compressa* cultivated in control condition (open circles) and with 10 μM copper (black circles) for 5 days. The level of oxygen is expressed in nanomoles per microliter per minute, and time in days. Symbols represent the mean value of three independent experiments ± SD. Different letters indicate significant differences (*P* < 0.05)

The level of produced oxygen under light (photosynthesis) decreased in control alga from 8.5 to 6.1 nmoles μL^− 1^ min^− 1^ whereas in treated alga it increased from 8.5 to 9.6 nmoles μL^− 1^ min^− 1^ at day 2, which represent a 32% of increase compare to control, and then decreased to 7.5 nmoles μL^− 1^ min^−^ 1 at day 5, which represent a 23% of increase compare to control (Fig. 3a). The level of consumed oxygen in dark (respiration) in control algae was 1.7 nmoles μL^− 1^ min^− 1^ and did not change at day 1, but increased to 3.8 nmoles μL^− 1^ min^− 1^ at day 5 whereas in treated alga it increased from 1.7 to 3.5 nmoles μL^− 1^ min^− 1^ at day 1 (106% of increase), and to 4.5 nmoles μL^− 1^ min^− 1^ at day 5 (18% of increase) (Fig. 3b). Thus, net photosynthesis and respiration increased in *U. compressa* exposed to copper excess. It is important to mention that oxygen produced in high light was higher than oxygen consumed in dark, which is in accord with level of normalized reads observed in kinetic analyses.

### Copper-induced increases in activities of enzymes involved in C, N and S assimilation

**Fig. 4:**
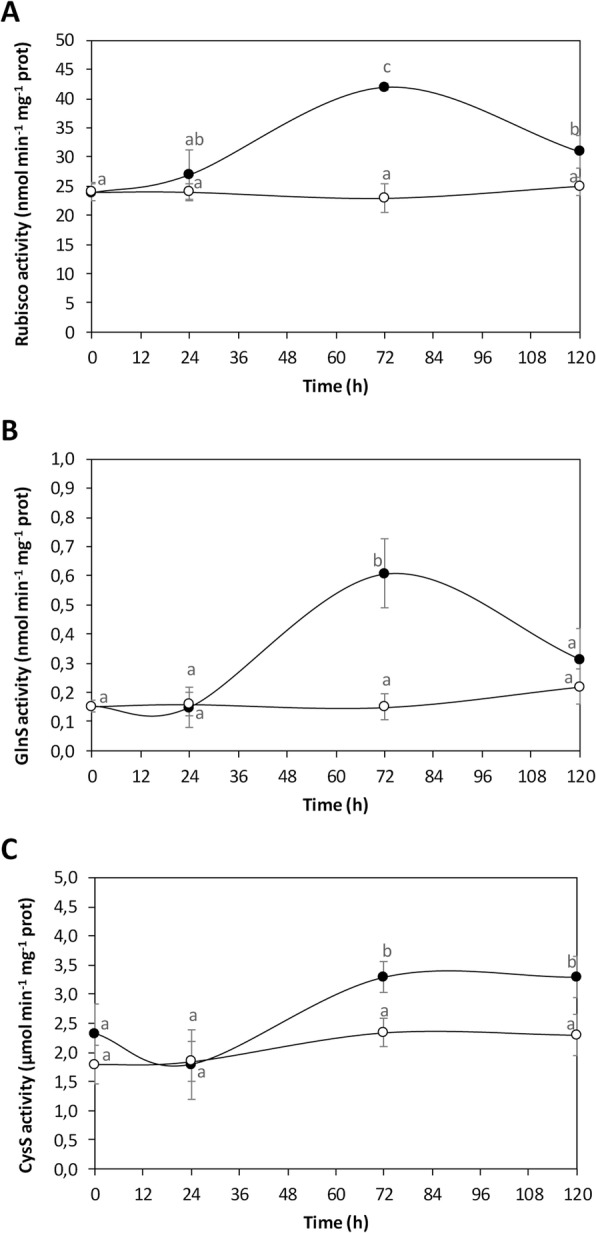
Activity of key regulatory enzymes involved in C assimilation, ribulose 1.5 biposphate carboxylase /oxygenase (rubisco, **a**), N assimilation, glutamine synthase (GlnS, **b**) and in S assimilation, cysteine synthase (CysS, **c**). The activity rubisco and GlnS is expressed in nanomoles per minute per milligram of proteins and the activity of CysS is expressed in micromoles per minute per milligram of proteins, and time in days. Symbols represent mean values of three independent experiments ± SD. Letters indicate significant differences (*P* < 0.05)

The activity of the enzyme rubisco in control algae was 2 μmol min^− 1^ mg^− 1^ of protein and it remained at this level until day 5 whereas in treated alga it increased to 4 μmol min^− 1^ mg^− 1^ of protein at day 3 and decreased to 3 μmol min^− 1^ mg^− 1^ of protein at day 5 (Fig. 4a). The activity of glutamine synthase increased in control alga from 0.15 to 0.22 nmol min^− 1^ mg^− 1^ of protein, and in treated alga it increased to 0.61 nmol min^− 1^ mg^− 1^ of protein at day 3 and then decreased to 0.1 nmol min^− 1^ mg^− 1^ of protein at day 5 (Fig. 4b). The activity of cysteine synthase in control algae increased from 1.8 to 2.3 μmol min^− 1^ mg^− 1^ of protein whereas in treated algae it increased to 3.3 μmol min^− 1^ mg^− 1^ of protein at days 3 and remained at this level at day 5 (Fig. 4c). Thus, the activity of key enzymes involved in C, N and S assimilation increased in *U. compressa* in response to copper excess. The activities of enzymes rubisco and cysteine synthase were higher than the activity of glutamine synthase, which is in accord with the level of normalized reads.

## Discussion

### Copper-induced increased expression of proteins involved in photosynthesis and in repair and protection of photosystems

In this work, we showed that the marine alga *U. compressa* cultivated with 10 μM copper for 5 days displayed an increased expression of transcripts encoding subunits of LHCII, PSII, cytb6f, LHCI, PSI and ATP synthase as well as proteins involved in repair of PSII and protection of PSI. It is important to mention that the level of transcripts encoding subunits of PSII, PSI, and proteins involved in PS repair and protection, showed higher increases compared with those observed in the alga cultivated with 10 μM copper for 0 to 24 h [8]. In this sense, the level of transcripts encoding subunits of PSII increased 2 Log2 FC in the alga cultivated with copper for 24 h whereas it increased 5.3 Log2 FC in the alga cultivated with copper for 5 days [8]. In particular, the level of transcripts encoding subunits PsbB and PsbW increased 2 and 3.3 Log2 FC, respectively, in the alga exposed to copper for 24 h, whereas the level of these proteins increased 4.5 and 5.3 times, respectively, in the alga cultivated with copper for 5 days [8]. The level of transcripts encoding the subunit of PSI, PsaA, increased 3.4 Log2 FC in the alga cultivated with copper for 24 h instead it increased 7.7 Log2 FC in the alga cultivated with copper for 5 days [8]. The level of transcripts encoding the chaperone MET1, involved in the replacement of oxidized PsbA, increased 3.4 Log2 FC in the alga cultivated with copper for 24 h whereas it increased 5.6 Log2 FC in the alga cultivated with copper for 5 days [8]. Thus, the expression of genes encoding proteins that constitute photosystems and those involved in repair and protection of photosystems was higher in the alga cultivated with copper for days compare with the alga cultivated with copper for hours.

Net photosynthesis increased in the alga cultivated with 10 μM copper for 1 to 5 days. This is in accord with the increase in the level of transcripts encoding proteins that constitute PS and those involved in repair and protection of PS. In this sense, photosynthesis also increased in the green macroalga *U. pertusa* cultivated with 4 μM of copper for 3 days and in *U. flexuosa* cultivated with 0.8 μM of copper for 8 days [6]. In addition, an increase in photosynthesis was observed in the red macroalga *P. haitiensis* cultivated with 1 μM of copper for 3 days and in the brown macroalga *E. siliculosus* cultivated with 0.8 μM of copper for 8 h [9–11]. Furthermore, it was determined that *U. compressa* cultivated 50 μM of copper for 7 days showed no sign of cell mortality [12]. Thus, *U. compressa* is more tolerant to copper excess than other green, red and brown marine macroalgae. In addition, it has been shown that *U. compressa* cultivated with 10 μM of copper for 0 to 7 days showed an increase in the level of superoxide anions beginning at day 3 and increasing until day 7 and that the production of superoxide anions occurred in chloroplats and mitochondria [12]. Thus, copper-induced oxidative stress may damage proteins present in photosystems and these proteins may be synthesized and replaced due to an increase in their expression. The kinetic analyses of the level of transcripts showed that the most increased transcripts encode the subunit PsbA of PSII which is highly susceptible to oxidation [21, 22], the protease MET1 involved in removing damaged subunit PsbA, the ATP-dependent protease FtsH1 and the ATP-independent protease Deg1 involved in degradation of damaged PsbA. In fact, the removing of oxidized PsbA and its degradation requires the concomitant action of FtsH and Deg proteases as well as other proteases such as Clp and SppA [23, 24]. Other higher expressed proteins were subunits LhcB4 and LhcB2 of LHCII, the iron-sulfur Rieske subunit PetC of cytb6f, and ABC1K1. Thus, subunit PsbA, proteins present in PSII, a subunit of cytb6f, and the kinase ABC1K1 are highly expressed proteins in response to copper stress and they may correspond to proteins that are more susceptible to oxidative damage as well as those required to remove and replace oxidized proteins.

### Copper-induced increased expression of proteins involved in respiration

*U. compressa* cultivated with copper excess showed an increase in expression of transcripts encoding subunits of mitochondrial complexes I and III, and a subunit of ATP synthase, as well as an increase in respiration. This is in accord with findings in the red macroalga *P. haitiensis* cultivated with 0.1 to 50 μM of copper for 3 days that showed an increase in respiration with 0.1 to 50 μM, but displayed an inhibition in photosynthesis over 1 μM copper [10]. The number of proteins involved in respiration that were overexpressed in *U. compressa* is lower than those involved in photosynthesis suggesting that respiration is less sensitive to copper-induced oxidative stress than photosynthesis. In this sense, it has been reported that respiration is less sensitive to copper- and chromium-induced oxidative stress due to the accumulation of superoxide anions in organelles of *Euglena gracilis* [25]. The increase in respiration observed in *U. compressa* under copper stress may increase the level of ATP that is required for ATP-dependent synthesis and degradation of proteins, fatty acids and chlorophylls, as it has been observed in plants under abiotic stress [26]. Several enzymes involved in the synthesis and degradation of protein, fatty acids, chlorophylls appeared to be overexpressed in transcriptomes of *U. compressa* cultivated for 10 μM copper for 5 days (data not shown) indicating the high turnover of proteins, fatty acids and chlorophylls is occurring in response to copper stress.

### Copper-induced increased expression of enzymes involved in C, N and S assimilation

The highest expressed transcripts encoding proteins involved in C, N and S assimilation were enzymes involved in the reduction of CO_2_, nitrate and sulfate as well as detoxification of ammonium and synthesis of glutathione. It is well known that enzymes involved in assimilation of C, N and S are regulated through thioredoxins and that the latter are regulated by cellular redox state [27]. Regarding C assimilation, it has been shown that the enzymes of the Calvin-Benson cycle G3PDH, PRK, FBP and sedoheptulose-1,7-biphosphatase are regulated by thioredoxins in plants [28] and the small and large subunit of rubisco, PGK and R5PE are regulated by thioredoxins in the green microalga *Chamydomonas reinhardtii* [29, 30]. Regarding N and S assimilation, it has been determined that glutamine synthase and adenosine-5′-phosphosulfate reductase can bind thioredoxins suggesting these enzymes may be regulated by these proteins [31]. On the other hand, it is well known that enzymes of the Calvin-Benson cycle enzymes and thioredoxins can be directly oxydized by Reactive Oxygen Species (ROS) such as hydrogen peroxide and superoxide anions [31]. As mentioned before, copper stress induced the accumulation of superoxide anions in *U. compressa*, beginning at day 3 and increasing until day 7 [12]. This wave of superoxide anions may directly oxidize and inhibit enzymes involved in C, N and S assimilation and/or thioredoxins. In order to restore the level of active enzymes, an increase in expression of the genes encoding enzymes involved in C, N and S assimilation, and thioredoxins (data not shown) was observed in the marine alga *U. compressa* in response to copper stress.

### Copper-induced decrease in expression of genes involved in protein synthesis and degradation, cell signaling, and replication and DNA repair

The levels of transcripts that decreased the most were those encoding proteins involved in protein synthesis and degradation, signal transduction, and replication and DNA repair. Regarding protein synthesis, the level of transcripts encoding ribosomal proteins L2, L15, L16, S12 and S17 decreased. The latter contrast with the level of transcripts that increased in the alga cultivated with 10 μM for 3 days corresponding to ribosomal L9, 10, 23, 28, 31, 35 and S12, 15 and 20 [32]. In addition, the levels of chaperone proteins Hsp40 and Hsp70 were decreased whereas the level of Hsp14 was increased in the alga cultivated with copper for 3 days [32]. Regarding protein degradation, the level of transcripts encoding RING protein 32 appeared to be decreased whereas that the level of RING protein RBX1 increased in the alga cultivated with copper for 3 days [33]. Regarding signal transduction proteins, the level of transcripts encoding calreticulin, several serine/threonine kinases, a MAPKKK and a myb-related protein decreased whereas the level of a calmodulin, a histidine kinase and a phospholipase A increased in the alga cultivated with copper for 3 days [32]. Thus, transcripts that are decreased in response to copper excess encode proteins that differ from those that are increased in response to the copper stress. Regarding the MAPKKK, it has been shown that *U. compressa* cultivated with 10 μM copper, and with inhibitors of the MAPK corresponding to ERK, JNK and p-38, for 6 h to 6 days, displayed mostly an increase in the level of transcripts encoding antioxidant enzymes [34] which suggests that MAPK pathways mainly inhibit the expression of genes encoding antioxidant enzymes. In this sense, the level transcripts of MAPKKK11 that decreased in response to copper stress (this work) also suggest that MAPK pathways mostly inhibit the expression of genes encoding antioxidant enzymes.

### The importance of S assimilation in *U. compressa* in response to copper stress

The activity of the enzyme cysteine synthase was higher than the activity of rubisco and much higher than the activity of glutamine synthase indicating the importance of S assimilation in *U. compressa*. In this sense, it has been shown that the alga exposed to increasing concentrations of copper for 0 to 12 days displayed an increase in the level of glutathione (GSH) and phytochelatins (PCs), which are sulfur containing peptides [15]. The nano-equivalents of thiol groups present in glutathione and phytochelatins correlated with the level of intracellular accumulated copper [15]. In addition, the level of transcripts of encoding MTs also increased in response to increasing concentration of copper [16]. Thus, cysteine-rich peptides and proteins are involved in copper accumulation and probably participate in the inhibition of copper-induced oxidative stress in *U. compressa* by sequestering copper ions [12]. Thus, increased S assimilation allowing an enhanced synthesis of thiol-rich peptides and the increased expression of thiol-rich proteins involved in sequestering copper ions may be essential for copper tolerance and accumulation in *U. compressa*.

## Conclusions

The marine alga *U. compressa* showed an increase in net photosynthesis and respiration as well as in activities of enzymes involved C, N and S assimilation in response to copper excess and these responses were due, at least in part, to an enhanced expression of genes encoding proteins involved in these processes. The combination of these responses may represent an exceptional mechanism of copper tolerance among photosynthetic organisms.

## Methods

### Alga and seawater sampling

*U. compressa* was collected in Cachagua (32° 34S′), a costal site with no history of metal pollution, during spring 2018 and transported to the laboratory at 4 °C inside a cooler. Algae were identified visually based on their phenotype and have been previously identified based on the sequence of 18S cDNA. Algae were rinsed three times with filtered seawater collected in Quintay (33° 12′S), a pristine site. Algae were cleaned manually and sonicated three min in an ultrasound bath (Branson, Danbury, CT, USA) in order to remove epiphytic bacteria and organic debris.

### In vitro cultures

*U. compressa* (100 mg of fresh tissue) was cultivated in seawater without copper addition (control, day 0) and with 10 μM CuCl_2_ for 1, 3 and 5 days under irradiance of 50 μmoles m^− 2^ s^− 1^ and a photoperiod of 12 h light:12 h darkness, and each sample in duplicate. All samples were washed with 2 ml of 100 mM Tris-10 mM EDTA pH = 7.0, twice for 10 min, in order to eliminate copper bound to algal cell walls. Samples were dried with paper, frozen in liquid nitrogen, and stores at − 80 °C.

### RNA extraction and preparation of cDNA libraries

Total RNA was extracted from samples cultivated for 0, 1, 3 and 5 days using EZNA total RNA kit (Omega Biotek, GA, USA). To this end, the alga (100 mg) was frozen in liquid nitrogen and homogenized in 1 mL of TRK buffer with 20 μL of β-mercaptoetahnol. The homogenate was centrifuged at 12,000 rpm for 15 min and the supernatant was recovered. The supernatant was mixed with ethanol 70% and transferred to HiBind RNA mini column and washed with RNA washing solutions I and II. Total RNA, was eluted with 50 μL of distilled water treated with DEPC. Total RNA was cleaned using GenJet RNA cleanup and concentration micro kit (Thermo, MS, USA). Total RNA samples were send to BGI Genomic Center (Shenzen, China) where RNA quality was checked, stranded and pair-ended cDNA libraries were prepared and sequencing was performed using an Illumina 4000 sequencer.

### De novo assembly and annotation

Reads obtained using Illumina sequencing were trimmed, cleaned and assembled using Trinity software at BGI Genome Center (Shenzen, China). Transcripts were translated into proteins using BlastX software and UniprotKB data base, annotated using an e-value of e^− 3^ or lower and classified according to Gene Ontology (GO) using OmicsBox software (Biobam, VA, Spain) and those having an e-value of e^− 3^ or lower were selected.

### Identification of differentially expressed transcripts

Cleaned reads were mapped against transcriptomes using Bowtie2 software and raw reads were counted using eXpress (version 1.5.1). Reads were then normalized to CPM units using Trinity’s script abundance_estimates_to_matrix.pl under default settings. Differentially expressed transcripts were identified using EdgeR at an FDR < 0.01, and Log2 Fold of Change > 1 for up-regulated transcripts and Log2 Fold of Change < − 1 for down-regulated transcripts. Differentially expressed transcripts were identified at times: 0 vs.1, 0 vs. 3, 0 vs. 5 days. Differentially expressed transcripts were visualized as a heat-map using Spearman’s correlation coefficient on transcripts and samples, and hierarchical clustering (Additional file 4: Figure S3A). All biological replicates were more similar to each other than to other samples (Additional file 4: Figure S3B). To identify down-regulated transcripts, the one hundred more down-regulated transcripts at each sample time point were identified and they were classified in different processes such as protein synthesis and degradation, fatty acid synthesis and degradation, DNA synthesis and degradation, replication and DNA repair, transcription, splicing, secondary metabolism, cell growth, among others.

### Kinetics of transcripts encoding proteins involved photosynthesis, respiration and C, N and S assimilation

Transcripts encoding proteins involved in photosynthesis and respiration and enzymes involved in C, N and S assimilation were selected based on the annotation and GO domain (biological processes). Reads of selected transcripts were normalized using TMM normalization method and they were analyzed using MEV software. Groups of transcripts showing similar temporal expression pattern were created and transcripts showing an increased expression were selected in these groups.

### Quantification of photosynthesis and respiration

Photosynthesis was quantified cultivating 25 mg of fresh tissue (FT) of *U. compressa* in 2 mL of seawater in an oxygraph chamber (Hansatech, Norfolk, UK). O_2_ production was detected for 10 min, under a light intensity of 425 μmoles m^− 2^ s^− 1^ and respiration was determined cultivating 25 mg of the alga (FT) in 2 mL of seawater in the oxygraph chamber, and O_2_ consumption was detected for 10 min in darkness.

### Preparation of protein extracts

One g of *U. compressa* (FT) was frozen in liquid nitrogen and homogenized in a mortar. Three mL of 100 mM phosphate buffer pH = 7.0 containing 5 mM β-mercaptoethanol were added and the homogenization was continued. The homogenate was centrifuged at 14,000 rpm for 15 min and the supernatant was recovered. Proteins in the supernatant were precipitated by addition of 0.6 g of ammonium sulfate per mL of extract and the mixture centrifuged at 15,000 rpm for 30 min. Protein pellets were solubilized in 200 μL of phosphate buffer pH = 7.0 containing 2 mM of β-mercaptoethanol and 20% glycerol. Final extracts contained around 3 mg mL^− 1^ of proteins and they were stored at − 80 °C.

### Detection of enzymes activities involved in C, N and S assimilation

Rubisco activity was detected as described in [35]. One mL the reaction mixture containing 100 mM Tris-HCl pH 8.0, 1 mM ribulose 1,5-biphosphate, 10 mM KHCO_3_, 20 mM MgCl_2_, 5 mM creatine phosphate, 3 mM ATP, 10 U phosphoglycerate kinase, 10 U glyceraldehyde 3-phosphate dehydrogenase, 10 U creatine kinase, 0.15 mM NADH and 30 μg of protein extract was used to detect rubisco activity. The decrease in absorbance at 340 nm due to consumption of NADH was detected for 3 min and activity was calculated using the extinction coefficient of NADH (ε = 6.2 mM^− 1^ cm^− 1^).

Glutamine synthase (GlnS) activity was detected as described in [36]. One mL of the reaction mixture containing 200 mM HEPES buffer pH 7.0, 50 mM L- glutamate, 5 mM hydroxylamine, 5 mM MgCl_2_, 20 mM ATP and 150 μg of protein extract was used to detect GlnS activity. The reaction was incubated at 37 °C for 1 h and stopped by addition of 1 mL mixture containing 0.7 M ferric chloride, 20% (w/v) trichloroacetic acid and 0.3 M HCl. The mixture was centrifuged at 7400 g for 5 min and the supernatant was recovered. The absorbance of the supernatant was detected at 540 nm and activity was calculated using the extinction coefficient of γ-glutamyl-hydroxamate (ε = 0.85 mM^− 1^ cm^− 1^).

Cysteine synthase (CysS) activity was detected as described in [37]. One mL of the reaction mixture containing 50 mM phosphate buffer pH = 7.5, 10 mM O-acetylserine, 2 mM Na_2_S, 30 mM DTT, and 50 μg protein extract was used to detected CysS activity. The reaction was incubated at 37 °C for 1 h and the reaction was stopped by addition of 0.5 mL of 20% (w/v) trichloroacetic acid. Cysteine was detected by addition of 100 μL of acetic acid and 200 μL of ninhydrin reagent. The mixture was placed in boiling water for 10 min, rapidly cooled in ice and 550 μL of 95% (v/v) ethanol were added. The absorbance of the mixture was determined at 560 nm and activity was determined using the extinction coefficient the compound formed by ninhydrin and cysteine (ε = 25 mM^− 1^ cm^− 1^).

### Statistical analyses

Significant differences in the detection production and consumption of oxygen and in activities of enzymes involved in C, N and S assimilation were determined using one-way ANOVA at 95% of confidence interval, followed by Tukey’s multiple comparison post-test using the statistical software Prism6 (Graphpad Sofware Inc., CA, USA).

## Supplementary information

**Additional file 1: Table S1.** Sequencing, pre-processing of reads and assembly of *Ulva compressa* transcriptomes.

**Additional file 2: Figure S1.** Pie-Chart of the percentage of proteins involved in different biological processes in *U. compressa.*

**Additional file 3: Figure S2.** Pie-Chart of the percentage of proteins having similarity with plant and animal proteins.

**Additional file 4: Figure S3.** Heatmap of differentially expressed transcripts in *U. compressa* exposed to copper excess.

**Additional file 5: Table S2.** List of all transcripts in time points 0 vs. 1, 0 vs.3, 0 vs. 5, their differential expression and the encoded protein.

**Additional file 6: Figure S4.** Scheme of chloroplast Light Harvesting complex II (LHCII), photosystem II (PSII), cytochrome b6f, photosystem I (PSI), Light Harvesting Complex II and ATP synthase (A). Scheme of mitochondrial complex I, II, III and IV and ATP synthase (B). Subunits showing an increased expression are highlighted in black.

## Data Availability

Reads have been deposited in SRA database (NCBI), the accession number is PRJNA557176, and they will be released on 24th august 2020. Experimental data is available at the on line repository: 10.6084/m9.figshare.9108491

## Abbreviations

APS reductase: Adenosine 5′-phosphosulphate reductase
CysS: Cysteine synthase
FBP: Fructose 1,6 biphosphatase
FBPA: Fructose biphosphate aldolase
FC: Fold of change
FT: Fresh tissue
G3PDH: Glyceraldehyde 3-P dehydrogenase
GlnS: Glutamine synthase
GSH: Glutathione
MTs: Metallothioneins
PCs: Phytochelatins
PGK: Phosphoglycerate kinase
PRK: Phosphoribulose kinase
R5PI: Ribose 5-P isomerase
rubisco: Ribulose 1,5- biposphate carboxylase /oxygenase
TK: Transketolase
