# Supplementary material for: Copper-induced concomitant increases in photosynthesis, respiration, and C, N and S assimilation revealed by transcriptomic analyses in Ulva compressa (Chlorophyta)
Source: BMC Plant Biol. 2020 Jan 15;20:25. doi: 10.1186/s12870-019-2229-5 (PMC6964094; doi:10.1186/s12870-019-2229-5)
Supplement: Supplementary file 1 — Additional file 1: Table S1. Sequencing, pre-processing of reads and assembly of Ulva compressa transcriptomes. [file 12870_2019_2229_MOESM1_ESM.pdf]

Supplementary Table 1. Sequencing, pre-processing of reads and assembly of *Ulva compressa* transcriptomes.

Libraries	Total Raw Reads	Reads after QC and Decontamination
AA05910 (0 h-1)	100,260,000	98,680,000
AA06575 (0 h-2)	100,260,000	98,660,000
AA06071 (24 h-1)	96,590,000	95,100,000
TT1_23 (24 h-2)	97,790,000	94,740,000
AA06107 (72 h-1)	94,770,000	93,490,000
AA06459 (72 h-2)	100,280,000	98,960,000
AA05880 (120 h-1)	100,280,000	98,880,000
AA06305 (120 h-2)	95,270,000	94,110,000
Transcriptome assembly		
Total transcripts	140,84	
Total bases	221,850,501	
Min sequence lenght	300	
Max sequence lenght	5,14	
Average sequence lenght	1,575	
N50 lenght	3,093	
N75 lenght	1,953	
N90 lenght	662	
N95 lenght		
(G + C)	56.30	
N	0%	
Transcriptome completeness		
Completeness	93.4%	
Complete CEG	283	
Complete and single copy	30	
Complete and multi-copy	253	
Fragmented CEGs	9	
Missing CEGs	11	
Total CEGs database	303	
