# Supplementary figures and images for: Copper-induced concomitant increases in photosynthesis, respiration, and C, N and S assimilation revealed by transcriptomic analyses in Ulva compressa (Chlorophyta)
Source: BMC Plant Biol. 2020 Jan 15;20:25. doi: 10.1186/s12870-019-2229-5 (PMC6964094; doi:10.1186/s12870-019-2229-5)

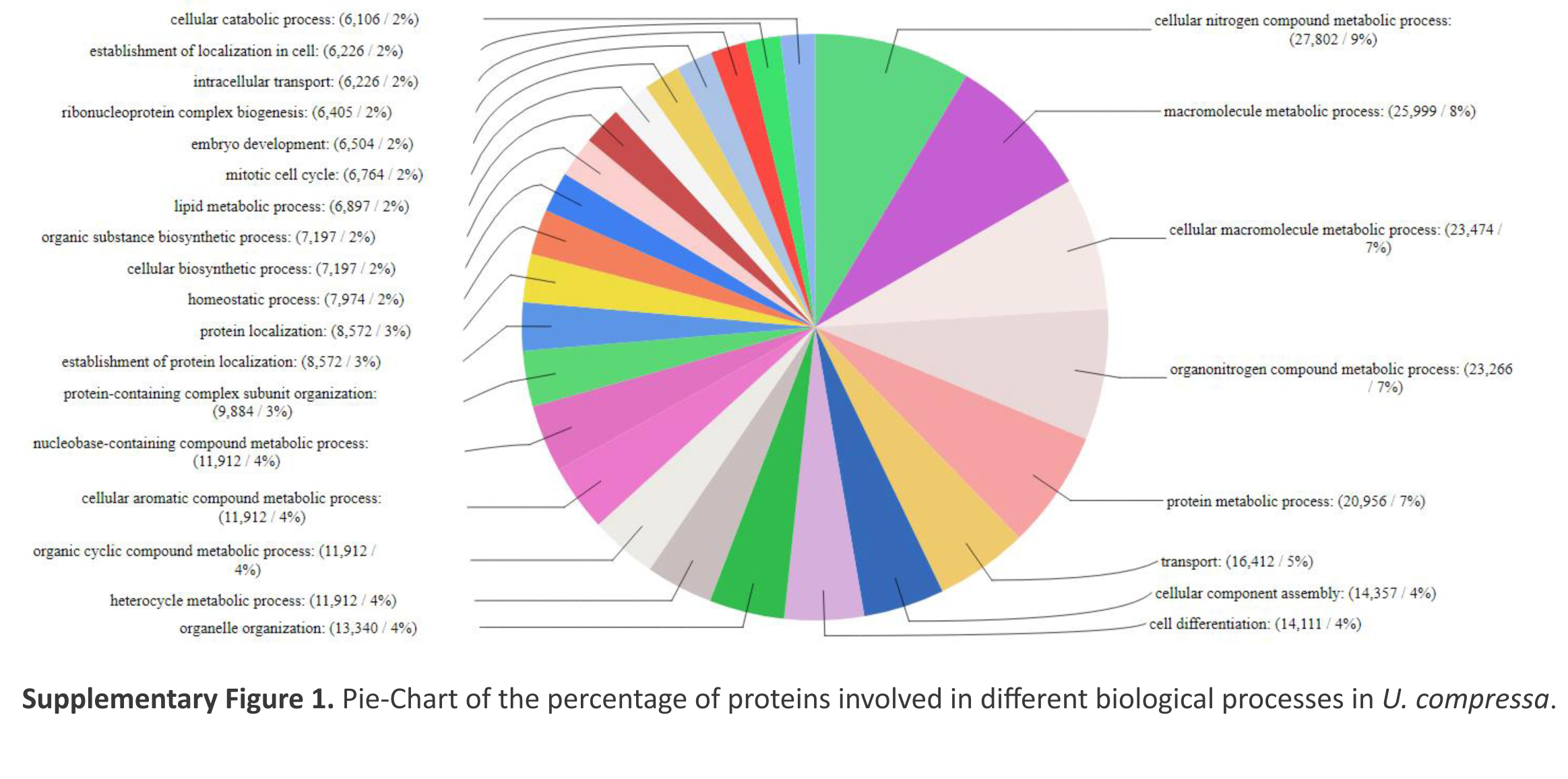

Supplement: Supplementary file 2 — Additional file 2: Figure S1. Pie-Chart of the percentage of proteins involved in different biological processes in U. compressa. [file 12870_2019_2229_MOESM2_ESM.tif]

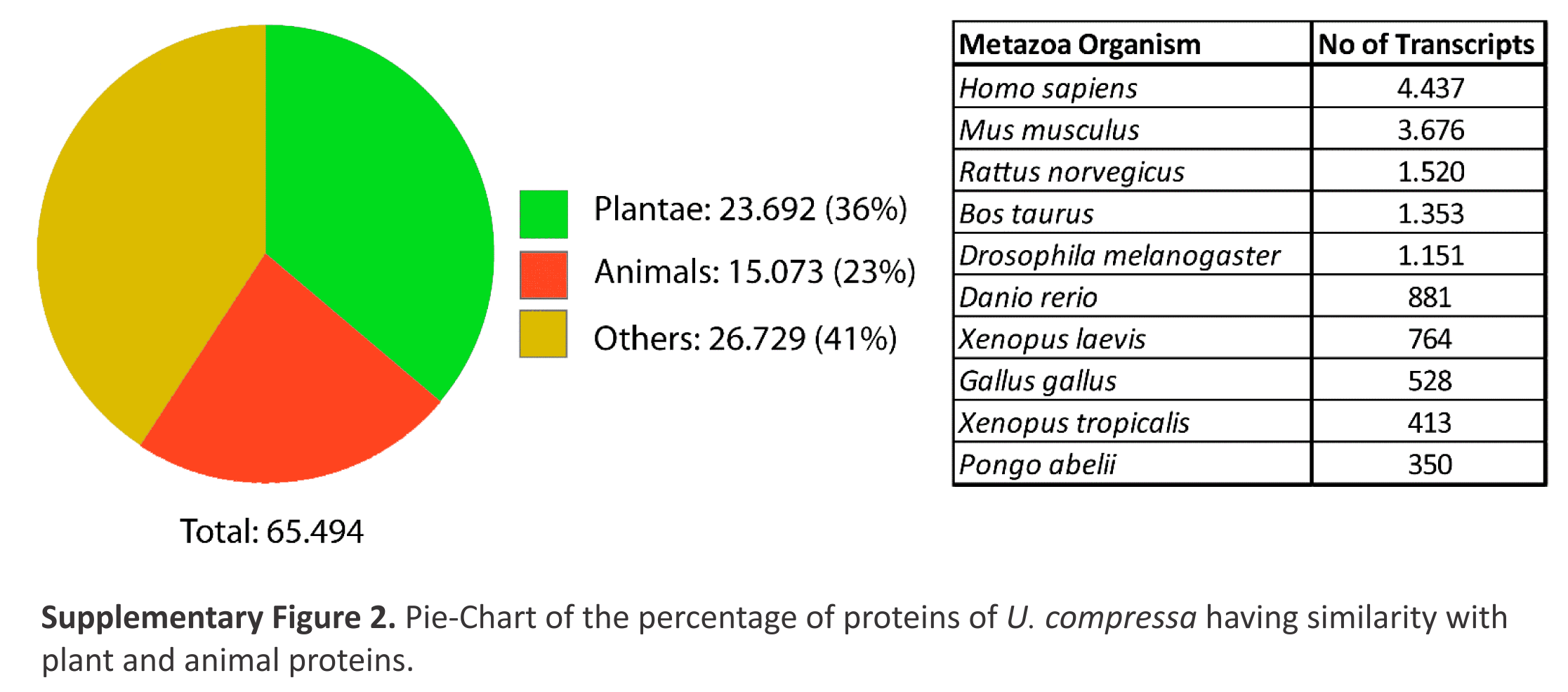

Supplement: Supplementary file 3 — Additional file 3: Figure S2. Pie-Chart of the percentage of proteins having similarity with plant and animal proteins. [file 12870_2019_2229_MOESM3_ESM.tif]

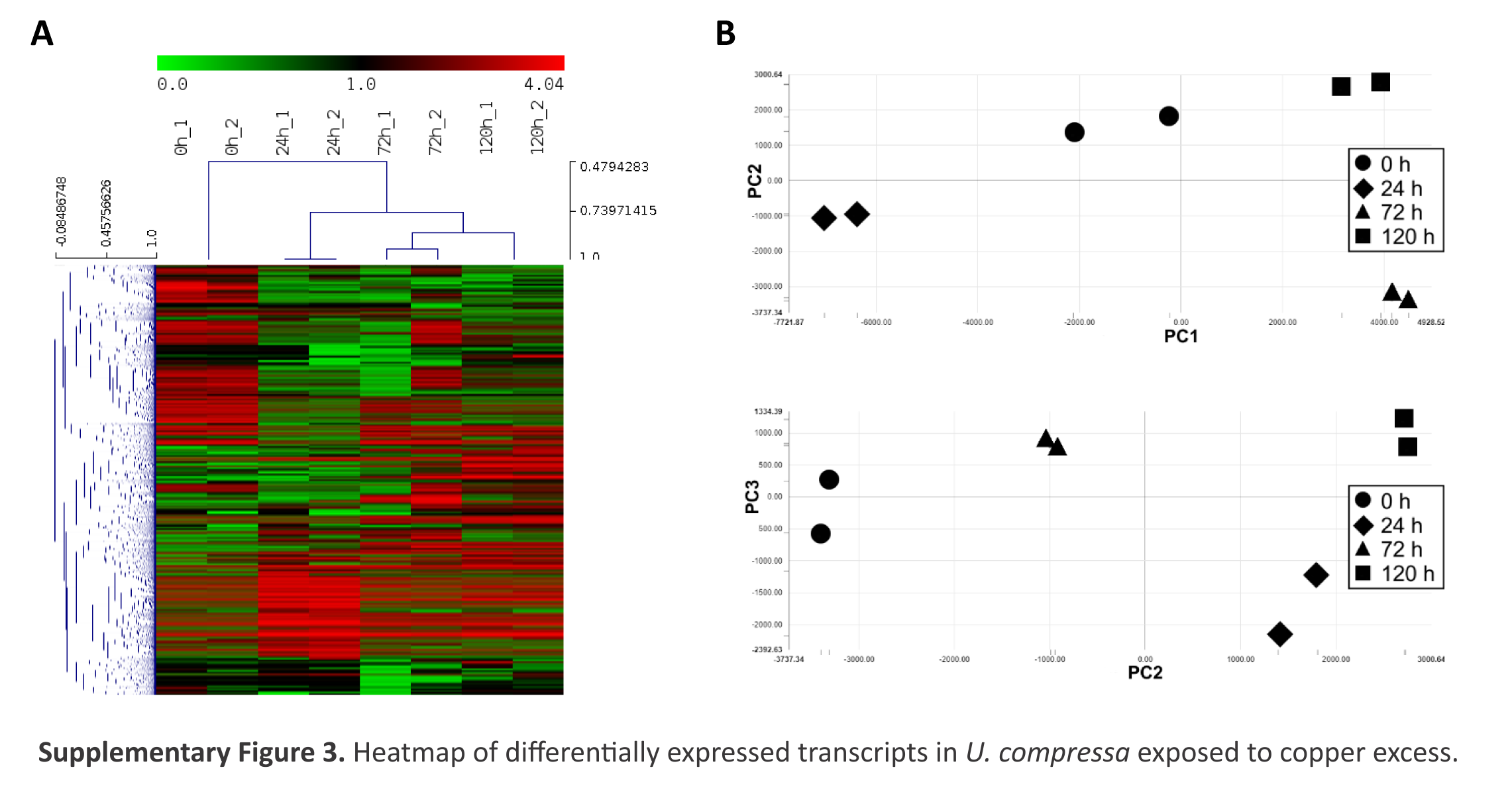

Supplement: Supplementary file 4 — Additional file 4: Figure S3. Heatmap of differentially expressed transcripts in U. compressa exposed to copper excess. [file 12870_2019_2229_MOESM4_ESM.tif]

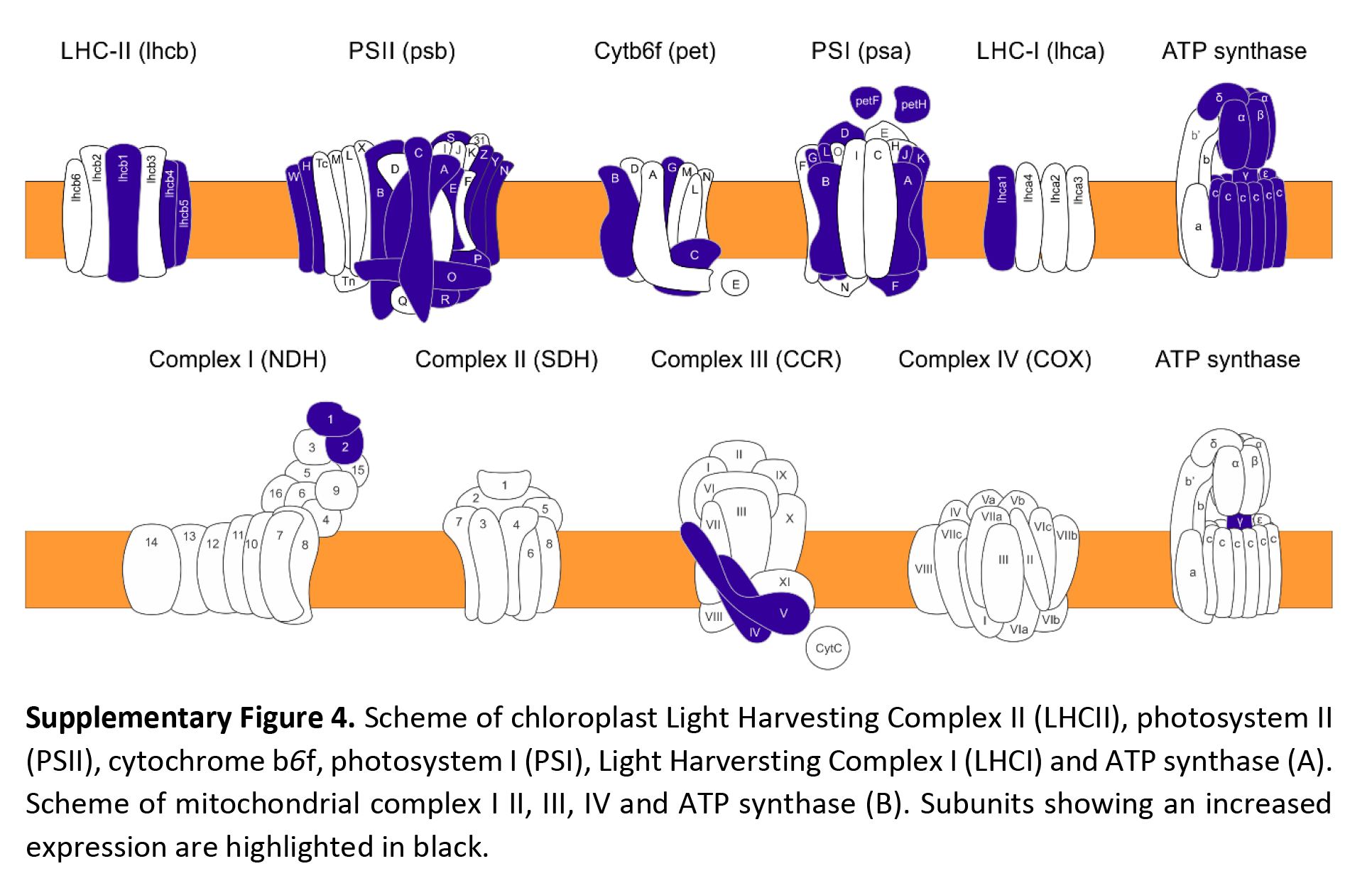

Supplement: Supplementary file 6 — Additional file 6: Figure S4. Scheme of chloroplast Light Harvesting complex II (LHCII), photosystem II (PSII), cytochrome b6f, photosystem I (PSI), Light Harvesting Complex II and ATP synthase (A). Scheme of mitochondrial complex I, II, III and IV and ATP synthase (B). Subunits showing an increased expression are highlighted in black. [file 12870_2019_2229_MOESM6_ESM.tif]
